# "Peer-Assisted Learning"(PAL) in the Skills-Lab – an inventory at the medical faculties of the Federal Republic of Germany

**DOI:** 10.3205/zma000952

**Published:** 2015-02-11

**Authors:** M. Blohm, J. Lauter, S. Branchereau, M. Krautter, N. Köhl-Hackert, J. Jünger, W. Herzog, C. Nikendei

**Affiliations:** 1 University of Heidelberg, Centre for Psychosocial Medicine, University Hospital for General Internal and Psychosomatic Medicine, Heidelberg, Germany; 2 University Hospital Heidelberg, Department of General Practice and Health Services Research, Heidelberg, Germany

**Keywords:** peer-assisted learning, tutors, Skills-Lab, simulation, medical education, practical clinical skills

## Abstract

**Background: **Over multiple years, the didactic concept of „peer-assisted learning“ (PAL) has proved to be valuable for medical education. Particularly in the field of the nowadays widely established Skills-Labs, the assignment of student tutors is both popular and effective. The aim of the underlying study is to assess the current status of PAL programs within German medical faculties’ Skills-Labs regarding their distribution, extent, structure and content based on a nation-wide survey.

**Methods: **All 36 medical faculties in Germany were contacted and asked for their participation (via telephone or in written form) in the survey encompassing 16 central questions as to the structure of established PAL programs. Data obtained were subject to quantitative and qualitative analysis.

**Results:** 35 of 36 (97.2%) medical faculties participated in the survey. A PAL program was shown to be established at 33 (91.7%) faculties. However, the results show distinct differences between different faculties with respect to extent and content of the PAL programs.

**Conclusions: **Among German medical Skills-Labs, PAL has been established almost ubiquitously. Further studies on the conception and standardization of training concepts appear to be pivotal for the advancement of PAL in the context of Skills-Labs.

## Zusammenfassung

**Hintergrund: **Das didaktische Konzept des „Peer-assisted learning“ (PAL) hat sich seit vielen Jahren in der medizinischen Ausbildung als wertvoll erwiesen. Insbesondere im Bereich der inzwischen weit verbreiteten Skills-Labs ist der Einsatz studentischer Tutoren gleichermaßen beliebt wie effektiv. Ziel des vorliegenden Artikels ist, auf Basis einer bundesweiten Befragung den aktuellen Stand über Verbreitung, Umfang und inhaltliche wie strukturelle Ausgestaltung der PAL-Programme in den Skills-Labs der deutschen Medizinischen Fakultäten zu erfassen.

**Methoden:** Sämtliche 36 bundesdeutsche Medizinische Fakultäten wurden kontaktiert und um Teilnahme an der 16 Leitfragen umfassenden Befragung zur Struktur von bestehenden PAL-Programmen im Skills-Lab-Bereich auf telefonischem oder schriftlichem Wege gebeten. Die erhaltenen Daten wurden quantitativ und qualitativ ausgewertet.

**Ergebnisse: **35 von 36 (97,2%) medizinischen Fakultäten nahmen an der Befragung teil. Ein PAL-Programm existiert an 33 (91,7%) Standorten. Die Ergebnisse zeigen, dass hinsichtlich Umfang und Inhalten jedoch große Unterschiede zwischen den Fakultäten bestehen.

**Schlussfolgerungen:** PAL ist an bundesdeutschen medizinischen Skills-Labs nahezu flächendeckend umgesetzt. Weitere Untersuchungen zur Konzeption und Standardisierung von Schulungskonzepten scheinen zentral für die Weiterentwicklung von PAL im Skills-Lab.

## Introduction

For many years, the didactic concept of „peer-assisted learning“ (PAL) has proved to be a valuable complement to academic staff teaching at universities. According to Boud et al. [1], the term “peer learning” comprises “learning with and from one another” and is defined as a mutually beneficial relationship and the sharing of knowledge. “Peer learning” may be carried out in various forms of social context (e.g. private, at school) and in different models of implementation [2]. In the underlying study, the term “PAL” is interpreted as the interaction in teaching and learning between tutors and tutees having a similar level of education [3]. Also within the context of PAL, there are distinct varieties with respect to its implementation [4], differing in number of tutors, number of tutees or gap in level of education. For example, tutors may originate from the same (“same-year PAL”) or a higher (“cross-year PAL”) academic year as their tutees [5].

In medical education, the concept of PAL has become deeply rooted and is carried out in various designs, whether in preclinical disciplines such as anatomy [6], in the context of problem-based learning (PBL; [7], [8]) or during the practical clinical teaching of physical examination [9], the training of communication [10] or procedural skills in so called Skills-Labs [11], [12], [13]. As to the latter aspect, it has been proven in several controlled trials that in many fiedls, PAL can be just as effective as classical academic staff teaching or even superior to it [11], [13], [14]. Weyrich et al. [13] showed, based on the results in OSCE (objective structured clinical examination [15]) trials after a teaching session in injection techniques, that students taught by peer tutors performed as well as tutees taught by academic staff, while both groups performed significantly better than controls who had not received any teaching.

Tolsgaard et al. [11] also compared the effect of classical faculty staff-led teaching to a PAL-based training of procedural skills. Here, tutees taught how to place a urinary catheter by peer tutors achieved even better results than the control group trained by academic staff. Using the example of a curricular clinical examination course, Hudson & Tonkin [14] were able to show that the interventional group having received peer tutor training performed on par with the control group having been taught by experienced physicians during an OSCE consisting of six stations including physical examination techniques and taking medical history.

These observations are most likely to be interpreted with respect to the cognitive and social congruence between tutors and tutees arising due to their similar level of education [3], [16]. An informal and relaxed learning atmosphere is created, facilitating the concession of shortcomings and posing questions [17], which is highly appreciated by tutees [14]. In addition to this, PAL teaching enables not only the participating tutees [18], but also the tutors, by means of their teaching activity, to develop their own abilities with regard to professional expertise, communication skills and assumption of responsibility [15], [19].

Hence, the concept of PAL offers the possibility to impart the clinical practical skills consented and proposed by the committee for practical skills of the Gesellschaft für Medizinische Ausbildung (Society for medical education) in a suitable context of the curriculum [20]. At the same time, tutors may acquire and deepen content of “learning how to teach”, as already laid down in the national competence-based catalogue of learning objectives (Nationaler Kompetenzbasierter Lernzielkatalog für Medizin (NKLM)) [21] and the Can MEDS [22].

In light of the above-described advantages of PAL and recent study data, the success of PAL, particularly in the context of Skills-Labs, the fact that it has become a pivotal part of clinical practical medical education does not come as a surprise. However, the extent of the implementation of PAL programs in German Skills-Labs still remains elusive. Furthermore, at present, nothing is known about potential differences between different medical faculties in regard to extent and respective infrastructure and content of this didactic concept. Based on a nation-wide survey, the aim of the present study is to provide an overview of the current status of PAL in German Skills-Labs. The main focus was laid on administration and organization, content of teaching and tutor training.

## Method

### Collection of data

Between June and July 2013, all 36 medical faculties in Germany were contacted via telephone and asked for their participation in the survey. In the attempt to find a suitable contact person for the subject of PAL, we first conducted an online search as to direct contact details of each Skills-Lab. If this proved unsuccessful, the dean’s office was asked to name a contact person. Only one person per faculty was interviewed. In the majority of cases, this was the medical director of the Skills-Lab, but also dean’s office staff, administrative staff of the Skills-Labs or student tutors. At the start of each telephone call, the interview partners were asked whether they preferred to participate via telephone or have the question form sent to them via e-mail to provide their answers in written form. In one case, relevant information was extracted from an informal e-mail from the contact person. In another case, information was merely derived from the Skills-Lab’s website, as personal participation had been declined.

#### Design of the questionnaire used for the nation-wide survey

The questionnaire was developed by four members of the Medical Faculty of the university of Heidelberg within the context of a focus group (n=4; 1 female, 3 male). All four persons had substantial expertise in the fields of administrative, practical and research-related aspects of the transmission of clinical practical skills and had all obtained methodological and didactic education (e.g. Master of Medical Education). In order to screen the designated questions for uniform understandability, three cognitive interviews were conducted. The final questionnaire comprised 16 central questions (with subitems, if necessary) on administrative-organizational, content-related and didactic aspects of PAL in the context of Skills-Labs. Both open and closed questions were used (please see the appendix for the full questionnaire). For increased interpretability and comparability, items related to questions on procedural skills and the medical specialties involved in their transmission were polled directly by means of pre-assigned checklists. Free text comments allowed for extending answers.

#### Data analysis

Where possible, analysis of the data was carried out indicating absolute frequencies, percentage quotations, mean values and range. As to mean values, arithmetic means were calculated. In the qualitative analysis, by forming suitable groups of similar answers, we tried to make a statement about the frequency distribution of the respective groups. The method used here was a frequency analysis according to Mayring [23], upon which categorical grouping and assignment of the keyword outlined programs was conferred. In terms of the definition of PAL, we considered the criteria to be met whenever student tutors were actively involved in teaching clinical practical skills. However, if student tutors exclusively worked in organizational and administrative fields, like in the maintenance of Skills-Lab rooms and material, this did not meet the criteria for PAL and data obtained in such a context was not included in the analysis.

## Results

### Sample

35 of 36 medical faculties agreed to participate in the present survey (35/36; 97.2%). At one faculty, suitable contact persons were only reached after several weeks and did not provide the completed question form but sent an informal e-mail from which data regarding the local status quo of PAL could be derived. Only one faculty explicitly declined taking part in the study (1/36; 2.8%). Seven (7/35; 20%) of the participating faculties were interviewed via telephone, while 28 (28/35; 80%) preferred to provide their answers in written form. As to the faculty that declined participation, it was possible to extract a lot of useful information from their well maintained website. Hence, the present results comprise data from all 36 German medical faculties on most of the relevant aspects (100%).

#### Distribution and dimensions of PAL programs in Skills-Labs

At 35 (35/36; 97.2%) medical faculties Skills-Lab training is part of the medical education and the only faculty which did not have a Skills-Lab is currently preparing to establish one. A PAL program within the Skills-Lab exists at 33 (33/36; 91.7%) medical schools with a percentage of female student tutors at 58.1% (Range 25-90). 28 interview partners provided answers on this aspect (28/33; 84.8%). Table 1 (Tab. 1) gives an overview of the dimensions and characteristics of the PAL programs.

#### Rationale for the implementation of a PAL program in Skills-Lab area

Regarding the motivation for an implementation of a PAL program, the details of 26 (26/33; 78.8%) faculties could be collected. Frequently mentioned reasons for the establishment included

1. the demonstrated effectiveness (through studies and internal evaluations),
2. the relief for or lack of medical teachers,
3. the development of practical clinical teaching, and
4. the possibility of targeted exam preparation by students for students (5 entries each for (1) to (4)).

The (5) implementation based on already established PAL programs of other medical faculties, (6) the facilitation of small group instruction, and (7) the formation out of a student initiative were each mentioned twice. Other reasons included, inter alia, the awakening of interest in teaching as well as cost efficiency.

#### Administrative framework

Regarding the administrative framework, data from 33 faculties (33/33, 100%) was obtained (see Table 2 (Tab. 2)). With regard to the management structures of the PAL programs, 30 faculties reported physician involvement in the management team, the remaining 3 faculties registered a purely student-run PAL program. Frequently mentioned disciplines (multiple answers possible) of the specialist directors of PAL programs included Anesthesiology / Emergency Medicine (twelve entries), internal medicine (five entries), surgery (four entries), general medicine (three entries), radiology and pediatrics (two entries). In eight cases, no specification of the field of study was made. In four cases, it was stated that the senior doctors had attained a MME (Master of Medical Education) as additional qualification (this was not explicitly assessed). Further occupations involved in management were employees in the areas of care (six entries, including four specialist nurses for anesthesia / intensive care medicine), education (four entries), psychology (four entries), Dean's Office staff (not further specified, two entries), sociology , social communication science, industrial engineering, midwife, theater education, medical assistant and simulated patients’ trainer (one entry each). On average, the Skills-Lab areas had two full-time employees a at time of survey (registration of 15 contacts to this aspect; 15/33; 45.4%). Four faculties reported student involvement in the management team (in addition to the three purely student-led PAL programs). Different forms of organization and administration are apparent: PAL areas that are centrally managed (22/32; 68.8%), that are in student co-management (19/32; 59.4%), or that are under the sole management of students (3 / 32; 9.4%). With regard to the funding base of the PAL program, details were provided by 27 of the contacts. 20 representatives reported faculty resources, 7 entries stated tuition fees/ quality assurance funding as their source of financial means.

The tutors were employed up to 5 years at the various locations with an average payment of 9.2 € per hour (range 7 - 17 € per hour; 26 contacts gave details on this aspect; 26/33; 78.7%). In regard to the aspect of evaluation of the tutors in relation to their work, 29 participants provided details (29/33; 87.9%). Five sites reported not to conduct regular evaluations of tutors in regard to their work. Further five medical schools only reported an evaluation of the tutors by the participants in the courses run by the respective students, but no evaluation of the tutors themselves. A total of 19 faculties stated that they regularly evaluate their tutors. The evaluations were conducted in the form of feedback sessions, team meetings, questionnaires, quality-assessment weekends, mentoring or mutual supervision during the courses.

#### Course content

31 Contact persons (31/33; 93.9%) answered the question on skills in which their tutors are trained (see Table 3 (Tab. 3)). One Skills-Lab representative stated that tutors were only implemented in physical examination courses. In addition to the predefined skills in the questionnaire, the various Skills-Labs could also list individual offers. Frequently, additional skills (at least three entries) included urinary catheter implementation (ten entries), lumbar puncture, subcutaneous / intramuscular injection (eight entries), central venous catheter implementation , hand hygiene / surgical washing (six entries), arterial puncture (five entries), communication techniques (e.g. diagnosis communication) (four entries), diagnostic radiology (four entries), plaster casting (three entries).

The subject areas, in which tutors are used, are also shown in Table 3 (Tab. 3). Again, additional subjects to those predefined in the questionnaire could be mentioned: Orthopedics (six entries), urology (four entries), hygiene (four entries), radiology (three entries), geriatrics (two entries), psychosomatic medicine / psychiatry, pharmacology and transfusion medicine (one entry each). 20 sites (20/33; 60.6%) offer separate tutor mediated courses in preparation for care and nursing internships, clinical traineeship and / or the final practical year.

#### Tutor training

Information on the extent and frequency of training sessions was collected from 22 contacts (22/33; 66.6%; see Table 4 (Tab. 4)). One faculty contact stated that the training of tutors in technical skills was not yet standardized. Another reported the training of new tutors through experienced tutors and the attendance of physician led courses. Two contacts listed to offer refresher-trainings if necessary which was implemented in the form of the renewed participation in basic training. Results revealed a significant variation in the later: the most frequent entries on the scope of basic training ranged from 10-20 hours but varied from about 3 to 50 hours. In this case, the content is often differentiated in didactic and technical training, partly supplemented by internships in existing courses and teaching practices. In some cases, the basic training is implemented as part of a block course at the weekend. In general, basic training is offered once per semester, often supplemented by ongoing technical training in specific content. Single annual training sessions or purely demand-driven training programs are less common.

## Discussion

The presented nation-wide survey shows through the almost comprehensive implementation and high acceptance of the Skills-Labs in Germany that PAL plays a central role in mediating clinical practical skills in medical faculties. Regarding the motives for implementing a PAL program, size, facilities and content focus considerable differences could be detected between the individual faculties.

In the above-mentioned reasons for the implementation of PAL programs in the faculties surveyed, the esteem of teachers for the work of student tutors and the recognition of the effectiveness of PAL [11], [13] is expressed. Proven effectiveness of PAL, the desire to increase practical teaching as well as targeted exam preparation were frequently cited as key motivation for the establishment of a PAL program in the Skills-Labs by the representatives. So far, however, there is no information in extant literature or suggestions regarding differential models for a didactically useful interaction of physician and tutor led Skills-Lab classes.

Meanwhile Skills-Labs are established for teaching procedural practical clinical skills at almost all medical faculties of the Federal Republic of Germany [24]. Here, we can look back on experience of over five years at the vast majority of faculties, at five faculties the Skills-Lab already has a tradition of over 10 years. At 33 of the 35 faculties with a Skills-Lab, student tutors are used in the sense of peer-assisted learning (PAL). Not only the positive attitude of students and student Skills-Lab tutors could be demonstrated impressively in several studies [12], [14], [25]. Weyrich et al. [12] also already showed in their study on the acceptance of tutees in relation to the PAL format that 82% of respondents found the lessons by student tutors to be sufficient and only 1% wished to be taught only by the physician lecturers. The tutors were generally given very good feedback. Moreover, in a focus group, the tutors evaluated their own work as satisfactory and reported a gain in competence. Here, they considered small group instruction with a tutor on four tutees ideal and also listed the positive feedback by the students as a strong motivational factor. In a recent paper on a voluntary, tutors led, clinical traineeship preparation course [25], showed high student satisfaction with this PAL offer, both on the part of the student through good global evaluation of the course as well as on part of the tutors, who saw similar benefits of PAL as in the previously described studies, and also felt personal and professional growth in competence. With the large number of on average 22.4 tutors per Skills-Lab, there is reason to believe that such a commitment in teaching is attractive for students and profits tutors in their personal and professional development [14], [20], [25]. According Dandavino et al. [26] working as a tutor trains communication skills and thus has a positive effect on the doctor-patient interaction.

With regard to the administrative and infrastructural framework, large differences between PAL programs at medical faculties become apparent. First and foremost, this concerns the size of the Skills-Labs and their PAL programs, though only details to staff levels or for tutors numbers were provided in the present survey. The facilities on premises and technical equipment were not entered in the questionnaire but were - although not consistently for the whole of Germany - recorded in a recently published study on Skills-Labs in the German-speaking area [27]. While the vast majority of the PAL programs have a medical director, other staff involved in management and organization is recruited from a wide array of professional groups. The number of tutors and their monthly workload, also varies with the size of the PAL program. Often tutors are also involved in administrative duties beside their capacity in teaching or manage the Skills-Lab and thus the PAL area, in three cases completely independently. The recruitment of tutors at the beginning of the clinical study section at the latest shows that long-term tenure is aimed at in the PAL program, if possible, offering advantages for the faculty and tutors since both benefit from the long experience.

The hourly wages paid under the PAL programs sometimes differ quite strongly, also resulting work hours as part of the training are not always paid. Appreciation of the tutors work and the desire for continuous improvement of the PAL program offer manifests itself in the regular evaluation through the questioning of tutors about their work, which is already being performed regularly at the majority of faculties. However, student evaluations seemed to be more common and are even mandatory in some federal states [28]. The background for the observed differences between the PAL programs in terms of size, personnel and technical equipment as well as remuneration and evaluation remains unclear. Here, further studies on possible influencing factors such as the size of the medical faculty, funding of the faculty and the PAL program, the duration of the PAL program and the individual commitment of respective teachers are needed.

On the part of the skills currently taught in PAL programs, the present study provides a detailed overview. It reveals that certain skills are most frequently taught by student tutors in the Skills-Lab. These include the laying of intravenous access, blood withdrawal or basic surgical techniques in sewing and nodes. In addition, however, strong individual foci are set in the areas of physical examination, communication skills or specialized procedural skills. Although there are first attempts to define important to be learned competencies in the Skills-Lab [29], it is still unclear which skills are particularly suited for learning in the context of PAL. Here, further systematic studies are needed. Based on the frequency of reported individual skills (and the associated broader experience in teaching by tutors), the present survey may provide preliminary insight, although the effectiveness should be examined separately in further controlled studies for the respective skills. The now often highly differentiated range of Skills-Labs offers with specially designed course packages for particular challenges (e.g. internships, practical year) provides an argument for a high level of organization and the good human and financial resources of many PAL programs on the one hand, but also underlines the need and the high demand under students for offers in which they can develop practical clinical skills. This is reflected in a recent study in which 19.8% of pre-clinical students participated in a clinical traineeship preparation course voluntarily [25]. Similar results were recently shown in an Austrian study in relation to PAL, which described the establishment of a compulsory course for teaching basic clinical and practical skills for freshmen [30]. The six-part course with two virtual and four practical units intended to counteract deficits in practical training at an early stage and to prepare students for future internships. The evaluation of this teaching concept achieved high satisfaction scores among participants, who perceived the course content to be relevant for their future careers and specified good theoretical and practical personnel learning outcomes.

Training of tutors appears to be an essential prerequisite for the success of PAL. Only substantive training conducted by experienced lecturers gives the tutors the necessary skills and compentence advantage for their work ahead with their fellow students, most of whom are in a similar stage of study. This is underlined by the fact that PAL related concerns expressed by tutees are mostly rooted in feared technical shortcomings of the tutors in comparison to medical faculty staff [14]. In addition to the professional, technical training, basic training in didactic teaching skills is required in order to ensure appropriate and competent teaching of procedural skills. This should include skills in group leadership, role playing, feedback and learning transfer didactics. Since the main objective, and thus also the particular strength of PAL lies in the structured transferation of skills, the procedural structuring of training is also of central importance. Only when processes are consistent, always presented under the same conditions, and clearly understandable and comprehensible for all participants, can these also be passed on in the desired manner. There is certainly room for improvement in the assurance of quality for teaching in the context of PAL as so far only few published concepts for structured training approaches exist [25], [31].

## Limitations

With regard to the data collected, it has to be considered that claims were only obtained from individual representatives of the Skills-Labs. The group of respondents is thus characterized by a large heterogeneity in terms of occupation and qualification. Nevertheless, to be able to ensure the comparability of information collected, persons interviewed in the present study had the greatest expertise and the deepest insight into the framework of the organization and thematic orientation of the programs in relation to the contents of the survey and according to the information of the medical faculties. With regard to the training situation of the tutors , no objective findings could be reported as content and scope of training differ greatly between the faculties. The present survey does not present a differentiated inventory of the financial resources of the respective PAL programms, this could only be inferred indirectly via the personnel costs.

## Conclusion

This nation-wide survey shows the almost comprehensive implementation of PAL programs at German universities, however, also highlights considerable variations in terms of size, facilities and content focus. Future studies should serve the development of concepts and models for the closer interaction of physician headed Skills-Lab lessons and tutor led Skills-Lab classes. In this context, the question of, for example, which skills are more or less suited for the PAL lessons would be of high interest. Moreover, to our knowledge no studies exist on the acceptance and adjustment of the faculty teaching staff regarding PAL in the Skills-Lab to this date.

## Acknowledgements

We would like to thank all involved PAL program contact persons and Skills-Labs for their kind participation in this survey and for their willingness to share their data for publication. We thank Anna Cranz for excellent translation of the manuscript.

## Competing interests

The authors declare that they have no competing interests.

## Figures and Tables

**Table 1:**
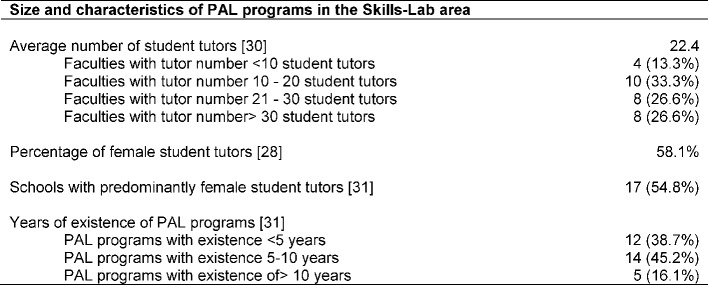
Size and characteristics of PAL programs in the Skills-Lab area; Specifying absolute numbers (n) and percentages (%); in square brackets number of faculties which provided information for the particular aspect with respect to faculties with PAL programs in the Skills-Lab area (n=33)

**Table 2:**
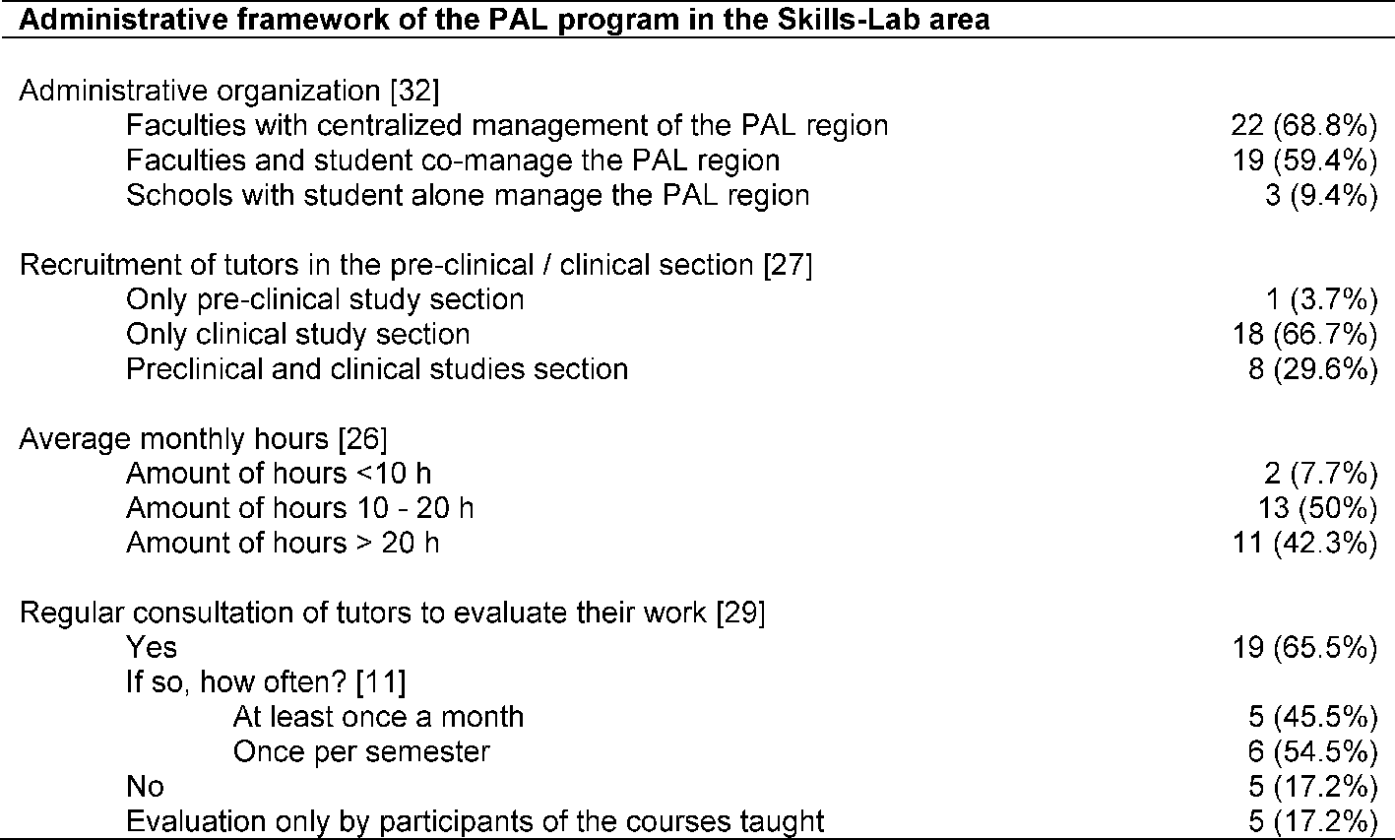
Administrative framework of the PAL program in the Skills-Lab area; Specifying absolute numbers (n) and percentages (%); in square brackets number of faculties which provided information for the particular aspect with respect to faculties with PAL programs in the Skills-Lab area (n=33)

**Table 3:**
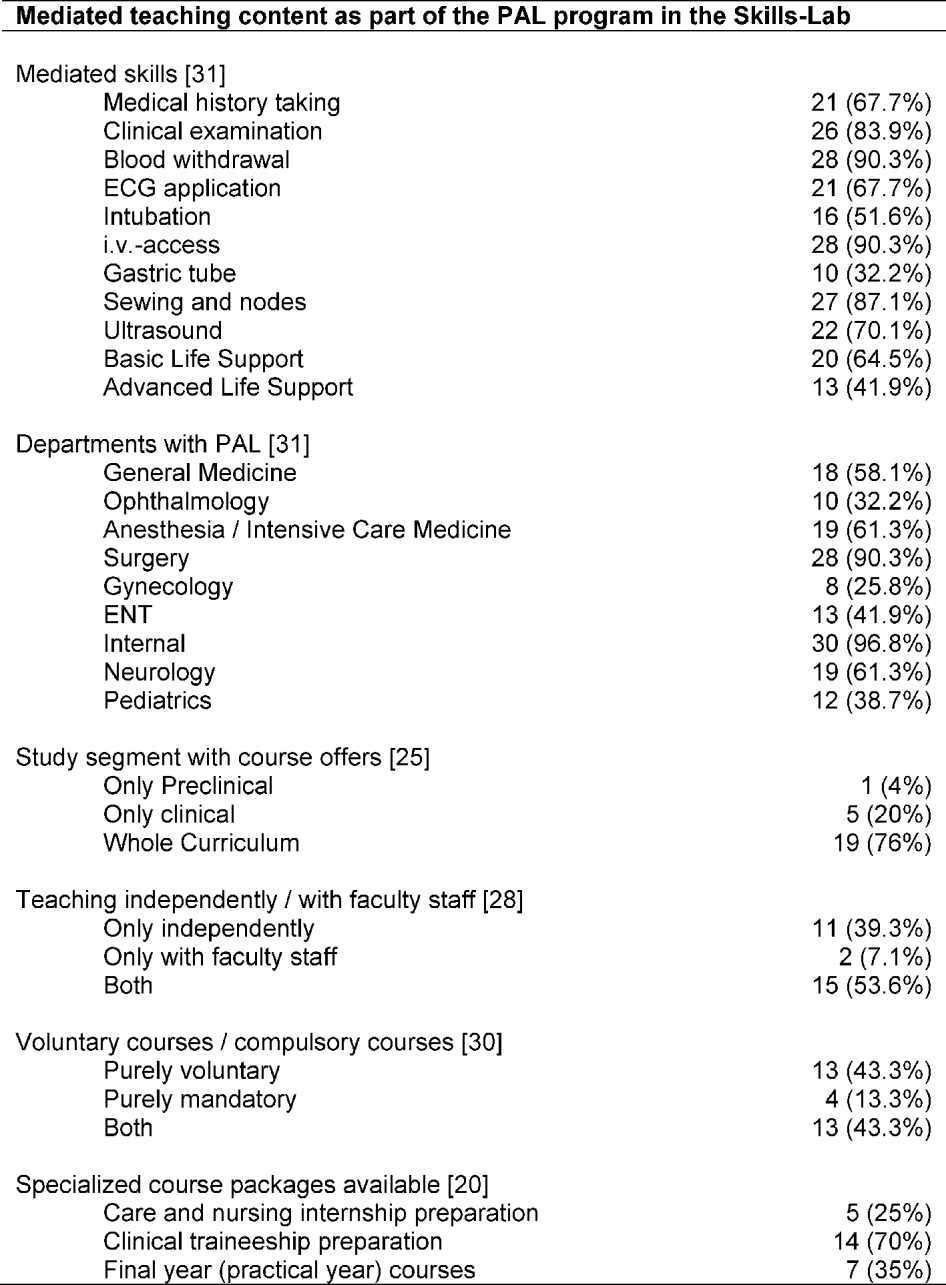
Mediated curricula as part of the PAL program in the Skills-Lab; Specifying absolute numbers (n) and percentages (%); in square brackets number of faculties which provided information for the particular aspect with respect to faculties with PAL programs in the Skills-Lab area (n=33)

**Table 4:**
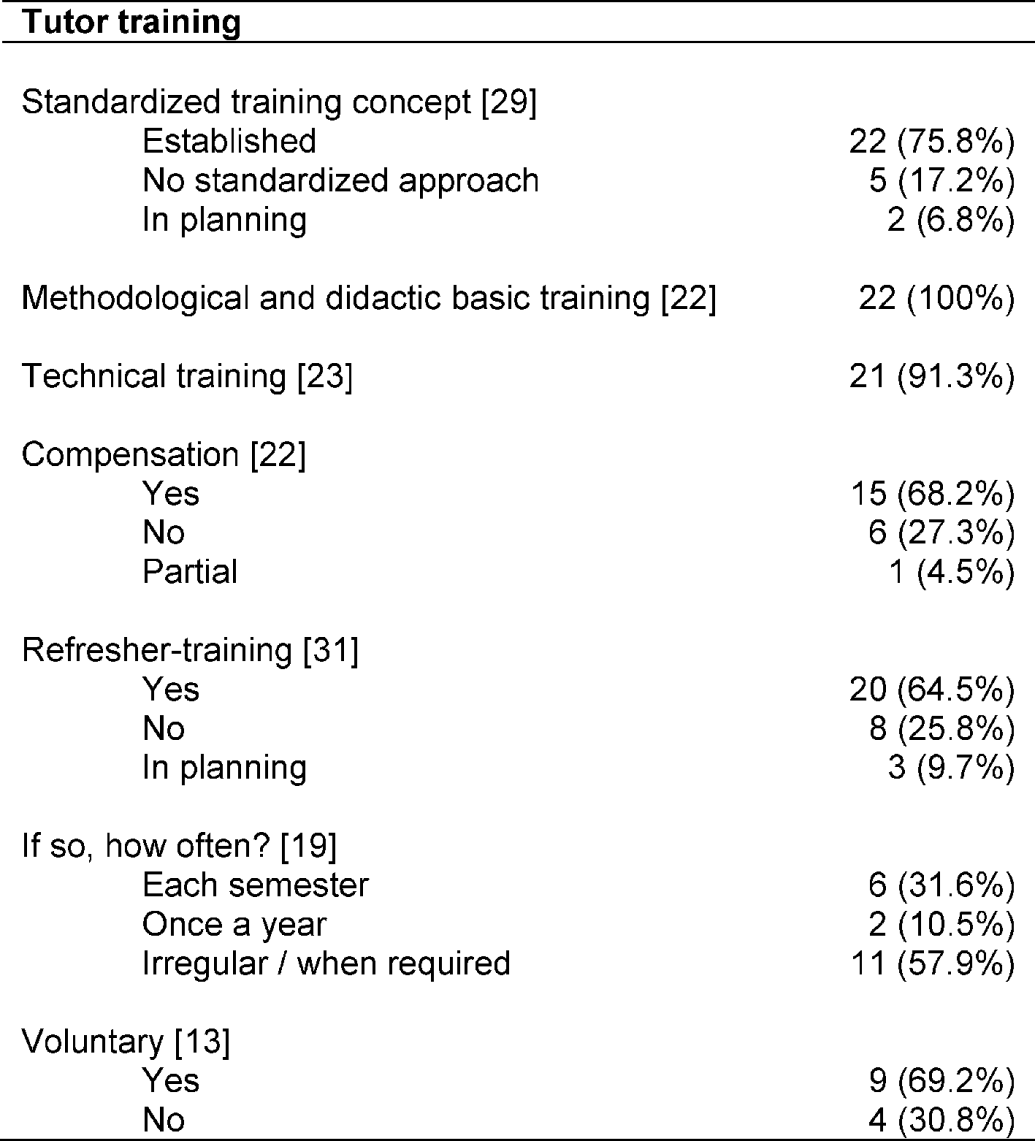
Tutors trainings; Specifying absolute numbers (n) and percentages (%); in square brackets number of faculties which provided information for the particular aspect with respect to faculties with PAL programs in the Skills-Lab area (n=33)

## References

[1] Boud D, Cohen R, Sampson J (2014). Peer learning in higher education: Learning from and with each other.

[2] Griffiths S, Houston K, Lazenbatt A, Baume C (1995). Enhancing Student Learning Through Peer Tutoring in Higher Education: A Compendium Resource Pack.

[3] Topping K, Ehly S (1998). Peer-assisted learning.

[4] Topping KJ (1996). The effectiveness of peer tutoring in further and higher education: A typology and review of the literature. High Educ.

[5] Hammond JA, Bithell CP, Jones L, Bidgood P (2010). A first year experience of student-directed peer-assisted learning. Act Learn High Educ.

[6] Nnodim JO (1997). A controlled trial of peer-teaching in practical gross anatomy. Clin Anat.

[7] Steele DJ, Medder JD, Turner P (2000). A comparison of learning outcomes and attitudes in student- versus faculty-led problem-based learning: an experimental study. Med Educ.

[8] Kassab S, Abu-Hijleh MF, Al-Shboul Q, Hamdy H (2005). Student-led tutorials in problem-based learning: educational outcomes and students' perceptions. Med Teach.

[9] Silbert BI, Lake FR (2012). Peer-assisted learning in teaching clinical examination to junior medical students. Med Teach.

[10] Nestel D, Kidd J (2005). Peer assisted learning in patient-centred interviewing: the impact on student tutors. Med Teach.

[11] Tolsgaard MG, Gustafsson A, Rasmussen MB, Hoiby P, Muller CG, Ringsted C (2007). Student teachers can be as good as associate professors in teaching clinical skills. Med Teach.

[12] Weyrich P, Schrauth M, Kraus B, Habermehl D, Netzhammer N, Zipfel S, Jünger J, Riessen R, Nikendei C (2008). Undergraduate technical skills training guided by student tutors. Analysis of tutors' attitudes, tutees' acceptance and learning progress in an innovative teaching model. BMC Med Educ.

[13] Weyrich P, Celebi N, Schrauth M, Moltner A, Lammerding-Koppel M, Nikendei C (2009). Peer-assisted versus faculty staff-led skills laboratory training: a randomised controlled trial. Med Educ.

[14] Hudson JN, Tonkin AL (2008). Clinical skills education: outcomes of relationships between junior medical students, senior peers and simulated patients. Med Educ.

[15] Waas V, Van der Vleuten C, Shatzer J, Roger J (2001). Assessment of clinical competence. Lancet.

[16] Lockspeiser TM, O'Sullivan P, Therani A, Muller J (2008). Understanding the experience of being taught by peers: the value of social and cognitive congruence. Adv Health Sci Educ Theory Pract.

[17] Schmidt HG, Moust JH (1995). What makes a tutor effective? A structural-equations modeling approach to learning in problem-based curricula. Acad Med.

[18] Santee J, Garavalia L (2006). Peer tutoring programs in health professions schools. Am J Pharm Educ.

[19] Sobral DT (2002). Cross-year peer tutoring experience in a medical school: conditions and outcomes for student tutors. Med Educ.

[20] Schnabel KP, Boldt PD, Breuer G, Fichtner A, Karsten G, Kujumdshiev S, Schmidts M, Stosch C (2011). Konsensusstatement "Praktische Fertigkeiten im Medizinstudium" – ein Positionspapier des GMA-Ausschusses für praktische Fertigkeiten. GMS Z Med Ausbild.

[21] Hahn EG, Fischer MR (2009). Nationaler Kompetenzbasierter Lernzielkatalog Medizin (NKLM) für Deutschland: Zusammenarbeit der Gesellschaft für Medizinische Ausbildung (GMA) und des Medizinischen Fakultätentages (MFT). GMS Z Med Ausbild.

[22] Frank JR, Danoff D (2007). The CanMEDS initiative: implementing an outcomes-based framework of physician competencies. Med Teach.

[23] Mayring P (1990). Qualitative Inhaltsanalyse. Grundlagen und Techniken.

[24] Kruppa E, Jünger J, Nikendei C (2009). Innovative teaching and examination methods--taking stock at German medical faculties. Dtsch Med Wochenschr.

[25] Blohm M, Krautter M, Lauter J, Huber J, Weyrich P, Herzog W, Jünger J, Nikendei C (2014). Voluntary undergraduate technical skills training course to prepare students for clerkship assignment: tutees' acceptance and tutors' training and attitudes. BMC Med Educ.

[26] Dandavino M, Snell L, Wiseman J (2007). Why medical students should learn how to teach. Med Teach.

[27] Segarra LM, Schwedler A, Weih M, Hahn EG, Schmidt, A (2008). Der Einsatz von medizinischen Trainingszentren für die Ausbildung zum Arzt in Deutschland, Österreich und der deutschsprachigen Schweiz. GMS Z Med Ausbild.

[28] Giesler M, Fritz H, Kadmon M, Stolz K, Wirtz HP, Biller S (2008). Lehrevaluation an den Medizinischen Fakultäten Baden-Württembergs. Z Evid Fortbild Qual Gesundhwes.

[29] Weitz G, Twesten C, Hoppmann J, Lau M, Bonnemeier H, Lehnert H (2012). Unterschiede zwischen Studenten und Ärzten im Anspruch an die praktische Ausbildung – Eine Bedarfsanalyse zum Skills-Training im Fach Innere Medizin. GMS Z Med Ausbild.

[30] Mileder L, Wegscheider T, Dimai HP (2014). Teaching first-year medical students in basic clinical and procedural skills--a novel course concept at a medical school in Austria. GMS Z Med Ausbild.

[31] Heni M, Lammerding-Köppel M, Celebi N, Shiozawa T, Riessen R, Nikendei C, Weyrich P (2012). Focused didactic training for skills lab student tutors - which techniques are considered helpful?. GMS Z Med Ausbild.

